# Effect of Intracanal Post Space Treatments on Push-Out Bond Strength of Fiber Posts to Root Dentin

**Published:** 2017-01

**Authors:** Hamid Kermanshah, Behnam Bolhari, Faraz Sedaghat, Ladan Ranjbar Omrani

**Affiliations:** 1 Associate Professor, Department of Operative Dentistry, School of Dentistry, Tehran University of Medical Sciences, Tehran, Iran; 2 Associate Professor, Department of Endodontics, School of Dentistry, Tehran University of Medical Sciences, Tehran, Iran; 3 Assistant Professor, Department of Endodontics, Dental School, Hamadan University of Medical Sciences, Hamadan, Iran; 4 Assistant Professor, Head of Dental Students Research Center, Department of Operative Dentistry, School of Dentistry, Tehran University of Medical Sciences, Tehran, Iran

**Keywords:** Post and Core Technique, Resin Cements, Root Canal Therapy

## Abstract

**Objectives:**

The main disadvantage of fiber posts is their low bond strength to root canal wall. The aim of the present study was to assess the effect of different root canal post space treatments on push-out bond strength of fiber posts to root canal dentin.

**Materials and Methods:**

After post space preparation in 40 endodontically treated human premolars, the teeth were randomly divided into four experimental groups: Group 1: control group, group 2: Endsolv R, group 3: ultrasonic cleaning, group 4: Clearfil Repair. Afterwards, the posts were bonded with Panavia F 2.0 bonding cement. The bond strength of fiber posts to root canal wall in the middle part of canal was evaluated following thermocycling using push-out test. Data were analyzed using one-way ANOVA and Tamhane’s multiple comparisons test. The failure mode of each group was determined under a stereomicroscope.

**Results:**

There was a significant difference in the mean push-out bond strength among the groups (P<0.05). The lowest bond strength was noted in the control group. The control group had significant differences with ultrasonic and Clearfil Repair groups (P<0.05). The bond strength of Endsolv R group increased; however, it was not significant (P>0.05).

**Conclusions:**

It seems that ultrasonic cleaning and Clearfil Repair can modify the root canal wall and significantly increase the bond strength of fiber posts.

## INTRODUCTION

Due to loss of structure in endodontically treated teeth, using intracanal posts may be necessary to increase retention of final restoration [1]. Recently, fiber posts have become more popular owing to some advantages over other intracanal posts, such as esthetic appearance [2], higher bond strength to root canal, closer elastic modulus to that of dentin and uniform occlusal stress distribution, leading to fewer, manageable root fractures [3–5].

Adhesion of fiber post to dentin is the result of bond between resin cement, intracanal post and root canal dentin [6]. Bond to root canal dentin is challenging due to difficult handling and delivery of adhesives into the root canal, anatomy of root canal and difficult cleaning of the root canal system [7–9]. An ideal bond to root canal is related to the quality of the hybrid layer and resin tags produced by resin bonding systems [10].

Self-etch priming systems are mainly used for bonding of fiber posts. These systems modify the smear layer and form a complex hybrid layer [11].

Root canal smear layer after post space preparation is completely different from the crown smear layer [12]. Scanning electron microscopic evaluations of root canals after post space preparation have shown that the root canal walls are covered with a thick smear layer containing rough debris and residues of gutta-percha and sealers [13]. This layer compromises effective infiltration of self-etch adhesives into the root canal dentin [14], and should be preferably removed before bonding of fiber posts. Several studies have been performed to evaluate the efficacy of different methods and irrigants for root canal smear layer removal such as ethylenediaminetetraacetic acid, ethylene acetate and ultrasonic irrigation [13,15].

Additionally, studies have shown that the sealers, based on their composition, may have negative impacts on bond strength [16–18]. Using resin sealers in endodontically treated roots can result in penetration of sealer into the dentinal tubules, forming resin tags [19]. This resinous layer that covers the root canal surface might prevent effective infiltration of adhesive into the root canal dentin [13]. To the best of authors’ knowledge, no previous study has attempted to modify this layer to increase the bond strength.

The aim of this study was to assess the efficacy of diamond coated ultrasonic tips and Endosolv R solution to remove resin sealer remnants from the root canal wall, and the effect of silane coupling agent on the bond strength of resin cement to root dentin rich in this resinous substance.

## MATERIALS AND METHODS

In this experimental study, 40 human premolars, with straight root canals and average root length of 15±1 mm were selected. All teeth were collected under a protocol approved by the Ethics Committee of Tehran University of Medical Sciences (code: 91-01-69-14064). Teeth were cleaned of calculus and soft tissues. After the storage of teeth in 0.1% chloramine T for one week, they were stored in distilled water at 4ܰC and were used within three months after their extraction [20]. The clinical crowns were cut 1mm above the cementoenamel junction with a low speed hand-piece and diamond disc (Degussa Dental, Hanau, Germany) under water cooling.

The canal preparation was performed using nickel-titanium rotary instruments (Profile; Dentsply Maillefer, Ballaigues, Switzerland). The preparation was conducted 1mm short of the apex and the prepared space was rinsed with 1–6mL of 0.2% chlorhexidine. The final irrigation was done with distilled water. Canals were dried with paper point (Ariadent, Tehran, Iran), and finally obturated with gutta-percha (Ariadent, Tehran, Iran) and AH26 sealer (Dentsply Maillefer, Ballaigues, Switzerland) by lateral compaction technique. The sealer was placed into the canal by a Lentulo spiral. Canal orifice was sealed with Cavite (Ariadent, Tehran, Iran), and the teeth were stored in distilled water at 37ܰC for one week.

After completion of the storage time, root filling material in the coronal 9mm of each canal was removed with peeso reamers #2 and 3 (MANI Inc., Tochigi, Japan), respectively such that at least 4mm of apical gutta-percha remained. The remaining gutta-percha was checked using radiography.

Teeth were randomly divided into four groups (n=10). In all groups, DT Light posts (RTD, St. Egreve, France), which are double tapered and made of epoxy resin (40%), and quartz fiber (60%), were bonded to the canal by Panavia F 2.0 dual-cure resin cement (Kuraray Co. Ltd., Osaka, Japan) according to the manufacturer’s instructions.

Group 1 (control): In this group, equal amounts of ED primers A and B were mixed and applied to intraradicular dentin with a microbrush and left for 30 seconds. Then, excess primer was removed with paper point and canal was dried with mild airflow. Afterwards, the same amounts of Panavia F 2.0 pastes (A and B) were mixed for 20 seconds and the fiber post was dipped in paste. Then, the post was completely seated into the canal with gentle finger pressure. Excess cement was removed, and the cement was light cured at the cervical root surface for 40 seconds using a Lava LED light curing unit (Ultradent Products Inc., South Jordan, UT, USA) with 1000 mw/cm^2^ intensity.

Group 2 (ultrasonic): Internal space of the canal was cleaned actively using E4D tip ((Varios, NSK Nakanishi Inc., Kanuma, Japan), which was mounted on an ultrasonic hand-piece (Varios 370, NSK Nakanishi Inc., Kanuma, Japan) with power setting on 7 for 20 seconds under tap water irrigation; then, the canals were irrigated and dried by paper point, and the posts were bonded into the canal as in the control group.

Group 3 (Endosolv R): A #50 K-file (Dentsply Maillefer, Ballaigues, Switzerland) was dipped in the Endosolv (Septodent, Cambridge, ON, Canada) and inserted into the canal. The file moved in in-and-out motion against the canal wall for about one minute to remove the sealer remnants. Then, the canal was irrigated for 20 seconds, dried by paper point and the posts were bonded as explained earlier.

Group 4 (Clearfil Repair): Silane coupling agent containing porcelain bond activator and Clearfil SE bond primer (Kuraray, Osaka, Japan) in equal amounts were mixed for 20 seconds. The mixture was applied to the root canal walls with a microbrush, left for five seconds and gently air-dried. The post was bonded to the canal as in the other groups.

After the bonding process, the roots were stored in 100% humidity at 37ܰ for one day. The teeth were thermocycled for 3,500 cycles between 5–55°C with 30 seconds of dwell time, and 15 seconds of transfer time. Each root was cut perpendicularly relative to its longitudinal axis by Isomet machine (Buehler, lake Bluff, IL, USA) and diamond disc under water cooling. Two horizontal cuts were made in the middle third region of each root. The thickness of each slice was 1±0.1mm.

The diameter of apical and coronal parts of root canal slices was measured using AutoCAD software 2006 (AutoDesk, San Rafael, CA, USA) by scanning photos of each slice. Subsequently, the cylindrical plunger was selected such that its size was 80–90% of the apical canal diameter. The plunger was positioned at the center of each post, while avoiding contact with the peripheral dentin and the force was applied in apico-coronal direction [15]. Load was applied at a crosshead speed of 0.5 mm/minute in a universal testing machine (Zwick, Ulm, Germany) until the fiber post was dislodged, and the maximum load at failure was recorded in Newtons (N). After bond failure, thickness of the slice was measured with a digital caliper. By dividing the force (N) by the bonding surface area of each slice (in square millimeters), push-out bond strength was calculated in megapascals (MPa).

$$\tau =\frac{N}{\pi \left({\text{r}}_{1}+{\text{r}}_{2}\right)\text{h}}$$

The failure mode of each slice was evaluated under a stereomicroscope (Nikon type 102; Nikon Corp., Tokyo, Japan) at x30 magnification to determine the percentage of each type of failure mode namely adhesive failure between dentin and resin cement, adhesive failure between post and resin cement, mixed failure, cohesive failure in intracanal post and cohesive failure in dentin. The data were compared using one-way ANOVA, followed by multiple comparisons by Tamhane’s test. The level of significance was considered as alpha=0.05.

## RESULTS

The mean and standard deviation of push-out bond strength values for different root canal post space treatments are presented in Table 1.

**Table 1:** The mean, standard deviations (SD), maximum, and minimum of push-out bond strength data (MPa) according to different pretreatment methods.

**Groups**	**Mean (SD)**	**Maximum**	**Minimum**
Control	4.6 (2.5) ^a^	13.34	1.63
Ultrasonic	10.6 (5.6) ^b^	25.01	3.07
Endosolv R	7.8 (4) ^ab^	15.80	1.21
Clearfil repair	9.2 (4.8) ^b^	18.66	2.54

The different superscripted letters indicate statistically significant differences (P<0.05).

Significant differences were noted in this regard among the groups (P<0.05). The control group had the lowest bond strength, followed by group 3 (Endosolv R), group 4 (Clearfil Repair) and group 2 (ultrasonic), respectively. The control group had a significant difference with groups 2 (P=0.002) and 4 (P=0.007). Group 3 had no significant difference with the other groups (P>0.05).

The failure modes were mainly adhesive in all groups; by increasing the bond strength, the frequency of adhesive failure mode decreased (Table 2).

**Table 2:** The percentage of modes of failure in each experimental group

Failure	Adhesive cement and dentin	Mixed	Adhesive cement and post	Cohesive post	Cohesive dentin
Mode Groups					
Control	59	33	8	0	0
Ultrasonic	45	45	10	0	0
Endosolv R	52	30	18	0	0
Clearfil repair	48	38	14	0	0

## DISCUSSION

The adhesive bond strength of fiber posts to root canal wall has been measured by a variety of methods [21]. These methods include microtensile, shear, pull-out, and push-out bond strength tests [22]. The push-out test is more reliable and reproducible than other methods [21]. One disadvantage of push-out test is non-uniform stress distribution. To overcome this problem, the sections should be prepared in 1mm thickness [21]. The plunger size must be selected for each slice according to root canal size in apical region; studies have shown that a plunger, which has a diameter slightly smaller than the canal diameter minimizes interfacial sliding friction [23].

In our experimental study, Panavia F 2.0 self-etching, self-priming adhesive resin cement was used, and push-out bond strength of fiber post to root canal wall was measured in the mid-root section. These sections were selected to eliminate the effect of root section variations such as decrease of tubular density and diameter in the apical region, lower penetration of light to the apical portion and apical calcification [11,24]. Sodium hypochlorite was not used as an irrigant solution in this study to avoid its possible negative effects on bonding process and polymerization of resin cement [24].The results of this study revealed a significant increase in the bond strength of fiber post to root canal, when ultrasonic cleaning was performed and also when silane coupling agent was applied to the canal.

Providing adequate bond between root canal wall and fiber post is very important for survival of restorations [25]. When using self-etching/self-priming adhesive systems, removal of smear layer and opening of dental tubules is not recommended, as they bond to the superficial layer of dentin via the smear layer. Nevertheless, the thick smear layer produced by drilling during post space preparation might prevent effective etching of ED primer II [26]. ED primer II contains 10-methacryloyloxi-decyl-dihydrogen-phosphate monomer, which is a weak acid and may not be able to dissolve the thick root canal smear layer. It seems that ultrasonic cleaning by diamond-coated tips mechanically removes this smear layer and subsequently induces a normal smear layer, which is suitable for proper bonding of adhesive system. Many studies have evaluated the effectiveness of ultrasonic agitation on smear layer removal from the root canal wall [27,28]. Srirekha et al. [13] used passive ultrasonic agitation with different irrigants to remove root canal smear layer after post space preparation. Their results showed that regardless of the type of irrigant solution, smear layer was effectively removed in contrast to the control group. Another study revealed that dentin surface prepared by ultrasonic device displayed greater contact surface for bonding process [29]. However, Lacerda et al. [30] used CVD diamond ultrasonic tips following irrigation of root canal with different solutions for root canal cleaning, but their method did not increase fiber post bond strength. This difference may be related to the use of different devices.

In our study, the bond strength of fiber posts increased in the Endosolv R group; however, it was not significant. Endosolv R is a combination of phenyl ethylic alcohol and, formamide; this solution helps remove phenolic resin-based sealers in retreatment of endodontically treated teeth [30]. Studies have shown that Endosolv R is effective in softening and removing of different resinous sealers from root canals [31,32]. In one study, Endosolv R increased microtensile bond strength of self-etch adhesive resins to AH plus contaminated dentin [33]. Nonetheless, no study has used Endosolv R before bonding of fiber posts. Presumably the selected time for Endosolv R in this study was not sufficient to remove the sealer; increasing the application time might significantly increase the bond strength of fiber post to dentin.

In the Clearfil Repair group, bond strength increased significantly in comparison to the control group in our study. Clearfil Repair is a fifth generation adhesive system, which has been produced for intraoral repair of fractured porcelain or composite restorations [34]. This system contains silane in its composition, which probably enhances the wetting ability and deeper penetration of adhesive into the root surface covered by resinous sealer [34,35]. Additionally, it seems that silane acts as a coupling agent, and makes a covalent bond with epoxy resin in AH26 sealer [35];it also forms a siloxane bond (- Si-O-Si-) with the silanated silica fillers in Panavia F 2.0 cement. Therefore, the bond strength increases [36]. Clearfil Repair kit has been effective for composite repair in many studies; although it has not been used in the root canal system in the literature [34,37,38].

According to the limitations of this in vitro experimental study, it can be concluded that despite the complexity of bonding of intracanal posts to root canal walls, method of root canal pretreatment has a significant effect on bond strength of self-etch resin cements to dentin. Further studies are required to assess the durability of bonds by mechanical loading.

## CONCLUSION

On the basis of the results obtained in this study, it can be concluded that ultrasonic cleaning and Clearfil Repair system are suitable pretreatment methods for increasing the bond strength of fiber posts to root canal wall.
